# Masked volume wise principal component analysis of small adrenocortical tumours in dynamic [11C]-metomidate positron emission tomography

**DOI:** 10.1186/1471-2342-9-6

**Published:** 2009-04-22

**Authors:** Pasha Razifar, Joakim Hennings, Azita Monazzam, Per Hellman, Bengt Långström, Anders Sundin

**Affiliations:** 1 Molecular Imaging & CT Research, GE Healthcare, SE-53188 Waukesha, Wisconsin, USA; 2 Uppsala Applied Science Lab (UASL), GE Healthcare, Uppsala Sweden; 3 Department of Surgery, Uppsala University Hospital, Uppsala, Sweden; 4 Department of Biochemistry and Organic Chemistry, Uppsala, Sweden; 5 Department of Radiology, Karolinska University Hospital, Stockholm, Sweden; 6 Department of Radiology, Uppsala University Hospital, Uppsala, Sweden

## Abstract

**Background:**

In previous clinical Positron Emission Tomography (PET) studies novel approaches for application of Principal Component Analysis (PCA) on dynamic PET images such as Masked Volume Wise PCA (MVW-PCA) have been introduced. MVW-PCA was shown to be a feasible multivariate analysis technique, which, without modeling assumptions, could extract and separate organs and tissues with different kinetic behaviors into different principal components (MVW-PCs) and improve the image quality.

**Methods:**

In this study, MVW-PCA was applied to 14 dynamic 11C-metomidate-PET (MTO-PET) examinations of 7 patients with small adrenocortical tumours. MTO-PET was performed before and 3 days after starting per oral cortisone treatment. The whole dataset, reconstructed by filtered back projection (FBP) 0–45 minutes after the tracer injection, was used to study the tracer pharmacokinetics.

**Results:**

Early, intermediate and late pharmacokinetic phases could be isolated in this manner. The MVW-PC1 images correlated well to the conventionally summed image data (15–45 minutes) but the image noise in the former was considerably lower. PET measurements performed by defining "hot spot" regions of interest (ROIs) comprising 4 contiguous pixels with the highest radioactivity concentration showed a trend towards higher SUVs when the ROIs were outlined in the MVW-PC1 component than in the summed images. Time activity curves derived from "50% cut-off" ROIs based on an isocontour function whereby the pixels with SUVs between 50 to 100% of the highest radioactivity concentration were delineated, showed a significant decrease of the SUVs in normal adrenal glands and in adrenocortical adenomas after cortisone treatment.

**Conclusion:**

In addition to the clear decrease in image noise and the improved contrast between different structures with MVW-PCA, the results indicate that the definition of ROIs may be more accurate and precise in MVW-PC1 images than in conventional summed images. This might improve the precision of PET measurements, for instance in therapy monitoring as well as for delineation of the tumour in radiation therapy planning.

## Background

Adrenal tumours are common incidental radiological findings in computed tomography (CT) examinations that are performed because of non-adrenal disease. For these adrenal tumours the term generally used is incidentaloma. As a result of the more frequent use and improved spatial resolution of modern multi-detector CT scanners, incidentalomas are being found more often. Because of their malignancy potential and because these tumours may cause hormonal hypersecretion, the discovery of an incidentaloma necessitates biochemical and radiological work-ups. Larger tumours (> 3–4 cm), tumours with high degree of suspicion of malignancy, and hormone-hypersecreting lesions are surgically removed [1-5].

The initial work-up for adrenal incidentalomas is based on laboratory testing and CT or Magnetic Resonance Imaging (MRI) examinations or both [2,3]. However, after CT or MRI examination or both, approximately one-third of adrenal tumours remain uncharacterized. To either rule out or to diagnose malignant disease, radiological follow-up for tumour size assessment is performed repeatedly by CT or MRI. Additional sensitive and reliable imaging methods are therefore needed to manage the considerable clinical problem to differentiate hormone-overproducing or malignant incidentalomas or both, for which treatment is needed, from benign non-hypersecreting lesions.

In previous studies, positron emission tomography (PET) with ^11^C labeled metomidate, [^11^C]-MTO (MTO-PET), has been introduced as a sensitive method to determine the adrenocortical origin of adrenal tumours [6-11]. [^11^C]-MTO has a high affinity for the ^11β^-hydroxylase enzyme in the adrenal cortex and MTO-PET can therefore differentiate adrenocortical tumours from those of non-adrenocortical origin.

Tumours larger than 1.5–2 cm and that are [^11^C]-MTO positive are generally readily depicted as a rounded, usually homogenously [^11^C]-MTO accumulating lesion. Detection is facilitated when the uptake in the lesion is higher or lower than the normal adrenal cortex; however, when the accumulation is similar in both, the tumour is generally recognized because it distorts the normal anatomy of the gland. Because of technical limitations related to camera resolution [12-14], the delineation of small adrenal tumours, less than 1–1.5 cm, by MTO-PET seems to be limited [7,8]. Moreover aldosterone-hypersecreting Conn-adenomas are usually in or below this size range, improvements are needed before PET can be used for lesion detection in primary aldosteronism.

For characterization purposes and to achieve reliable PET measurements of the tracer accumulation in tumours and normal tissues, MTO-PET may be performed dynamically with the examination started synchronously with the tracer injection and continued for 45 minutes. By dividing the scanning sequence into several short time frames, each typically one to a few minutes long, the [^11^C]-MTO accumulation in the tumour and the normal adrenal can be determined over time. The time frames representing the highest tracer uptake in the tumour, relative to the normal adrenal cortex, may then be summed to a so-called "summed image" to optimize the image reading. Dynamic MTO-PET images from 15 to 45 minutes after the tracer injection are typically summed to create transaxial images of regional radioactivity concentration. However, the summation of image data is generally performed routinely and is not based on a pharmacokinetic analysis of the tumour in the individual patient since such a procedure is time consuming. In addition to visualizing the lesion, it is essential for its characterization to perform accurate PET measurements by outlining regions/volume of interest (ROIs/VOIs) with high precision, especially when the tumour is small. It has previously been demonstrated that the magnitude and variation of the noise, and partial volume effect in the PET images might affect the precision of defining ROIs and the resulting SUV measurements [15].

In previous studies, Masked Volume Wise PCA (MVW-PCA) was introduced [16,17]. It was shown for several PET tracers that MVW-PCA could be used as a multivariate analysis technique, which without any kinetic assumption could extract and separate organs and tissues with different kinetic behaviors into different principal components (MVW-PCs). Moreover, MVW-PCA enhanced the image quality by improving the visual contrast between the anatomical structures thereby yielding more detailed anatomical information.

The aim of the present study was to apply the introduced MVW-PCA approach to MTO-PET examinations to obtain diagnostic information from the whole dataset. A further aim was to assess possible improvements in image quality achieved by applying MVW-PCA, which might lead to improvement of the precision when defining ROIs/VOIs used for PET measurements in tumour and normal adrenal gland.

## Methods

Here, MVW-PCA was applied to dynamic MTO-PET examinations of the adrenals of patients with small adrenocortical tumors (1–2 cm). Six of the seven patients had biochemically proven or suspected primary aldosteronism and one patient had a small hormonally silent incidentaloma. MTO-PET was performed before and 3 days after starting per oral cortisone treatment. The rationale behind this regimen was that possible variations in tracer uptake might be detected in the same patient during two different hormonal states. Moreover, each patient could function as his or her own control. The whole dataset, 0–45 minutes after the tracer injection, was used to include all information about tracer pharmacokinetics to extract and separate tissues with different kinetic behaviors in several uncorrelated pharmacokinetic components. The aim was three-fold: firstly, to evaluate the early phase of the MTO-PET sequence for additional information and possibly better depiction of small adrenocortical adenomas, (as compared to the visualization of these tumours in the later phases of the examination). Secondly, to determine the different MVW-PCs in the examinations and to compare the visibility of the tumours in these components with the summed images achieved by conventional summing of the dynamic PET sequence data 15 to 45 minutes after the tracer injection. Thirdly, to evaluate the SUVs in tumour and contralateral normal adrenal gland tissue for potential differences by drawing ROIs and including these tissues in the MVW-PCA images and the summation images, respectively.

### Tracer administration and patient data

MTO-PET of the adrenals was performed on seven patients, three male and four female, aged 34–78 years identified from a study performed by Hennings and co-workers [11].

The study was approved by the local ethics committee (No 02-262) and was performedin compliance with the Helsinki Declaration. Inclusion into the study was by informed consent. Each patient had a 1- to 2-cm adrenocortical adenoma as characterized by CT attenuation measurements. In addition to the initial study, MTO-PET was performed a second time on each patient after 3 days of per oral cortisone medication (dexametasone 0.5 mg three times daily). The patients were examined after a minimum of four hours of fasting, allowing free intake of clear liquids. Approximately 10 MBq/kg of [^11^C]-MTO per kilogram of body weight, corresponding to 715 ± 199 MBq (mean ± SD) range 370–1050 MBq, was administered. After a rapid intravenous bolus injection of the tracer, a 45-minute dynamic examination sequence was started which comprised 14 time frames: 5 × 1 minute, 5 × 3 minutes, 3 × 5 minutes and 1 × 10 minutes.

### Image analysis and PET measurements

#### Masked Volume Wise Principal Component Analysis

The method is based on using noise pre-normalized data that represent the whole scanned section of each frame as a single variable. They are applied after the background has been removed, i.e, masked out. The MVW-PC volumes that are generated contain organs and tissues with different and uncorrelated kinetic behaviors of the administered tracer, and the number of output MVW-PC volumes is equal to the number of PET frames of the study [16]. Since no region could be used as a reference region here, the kinetic pre-normalization was excluded from the MVW-PCA procedure. Only the first three components were explored since higher components contained only noise.

Software used for the application of MVW-PCA on dynamic PET images was developed in-house by one of the authors (PR) using Matlab 7.2 (The Mathworks, Natick, Massachusetts) with installed statistical and image processing toolboxes.

#### Region of interest

Both the summed images and the MVW-PC1 images were used to outline ROIs representing the adrenal tumour and the contralateral normal adrenal gland. Subsequently, 4 contiguous pixels corresponding to 0.64 cm^2 ^with the highest radioactivity concentrations, i.e., the "hot spot" [18] were identified in the tumour and in the normal contralateral adrenal gland, respectively. The coordinates for the individual pixels of these hot spots were recorded and used for calculation of SUVs.

Moreover, ROIs were defined using the isocontour function whereby the pixels with SUVs between 50 and 100% of the highest radioactivity concentration i.e., "50% cut-off" were delineated to outline the adrenal tumour and the contralateral normal adrenal gland respectively.

The mean Time Activity Curves (TACs) were calculated, plotted, and compared for ROIs delineated using the 50% cut-off method, which represented the adrenal tumour and the contralateral normal adrenal gland, respectively, in both the MVW-PC1 and the summed images. Furthermore, the radioactivity concentrations in the summed images were re-calculated to provide SUV images whereby the radioactivity concentration (Bq/cc) was divided by the injected dose per gram body weight.

To compare the precision by which the "hot spot" ROIs were outlined, using the two methods, the coordinates for the pixels representing the ROI "hotspots" in the summed images and the MVW-PC1 images, respectively, were imported into the corresponding SUV images. The SUVs for the corresponding pixels were registered and the mean SUV of the ROIs was calculated.

#### Statistical methods and illustrations

The mean SUV and the standard deviation of the mean (SEM) for the defined ROIs using both "hot spot" and 50% cut-off methods in the summed as well as MVW-PC1 images were calculated, respectively.

A univariate regression analysis was performed in which the SUVs obtained in ROIs defined in the MVW-PC1 images were plotted against the SUVs obtained in ROIs defined in the summed images.

Repeated measures ANOVA based on "Bonferroni's multiple comparison test", linear regression and Student's t-test (paired and two-tailed) were performed to investigate differences between the "hot spot" and the 50% cut-off methods when MVW-PC1 and summed images were used for delineation and quantification of the ROIs.

Here, Graph Pad Prism Version 4.03 (Graph Pad Software, Inc, San Diego, USA) was used to carry out all statistical analysis and graphical illustrations.

## Results

### Qualitative comparison

MVW-PCA identified three separate pharmacokinetic events in the dynamic MTO-PET examinations. The kidneys, pancreas, and spleen were clearly delineated in the MVW-PC2 images corresponding to the early and fast pharmacokinetic events in these organs. No qualitative diagnostic information about the depiction of the adrenocortical tumours could be retrieved from the MVW-PC2 component. A characteristic feature of [^11^C]-MTO is the slow, gradual accumulation of the radioactivity into the gastric juice. This intermediate pharmacokinetic event was isolated and displayed in the MVW-PC3 images which, however, were devoid of diagnostic information related to visualization of the adrenocortical tumours. MVW-PC1 corresponds to the kinetic with the largest variation and most predominant behavior of the [^11^C]-MTO in this study. This means that MVW-PC1 images contain organs and tissues with the largest and mostly preserved kinetic behavior during the whole scanning time. This component isolates the pharmacokinetic phase in the dynamic PET sequence, which best resembled the conventional summed images based on data 15 to 45 minutes after [^11^C]-MTO injection but the image noise was lower and the organ and tissue separation was better. In these images the adrenocortical tumours were readily depicted with qualitatively better lesion delineation than in the summed images.

**Figure 1:**
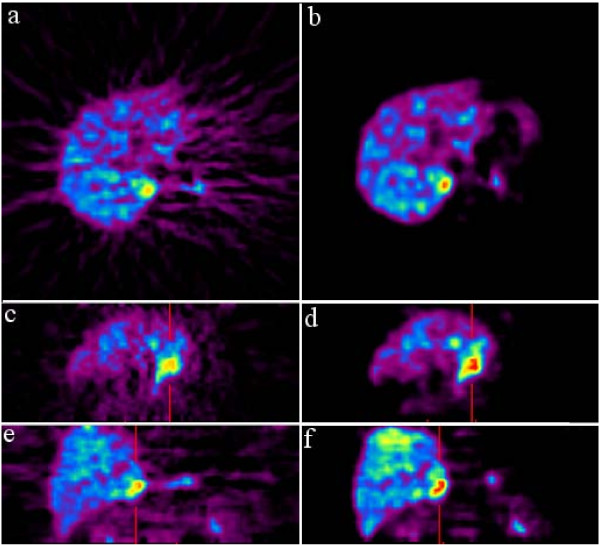
**First Column shows the summed images generated by summing images through frame 9–14 (15–45 min) and second column shows the corresponding MVW-PC1 images generated by application of MVW-PCA on the whole imaging sequence of 0–45 minutes**. These are shown in the transaxial (first row) sagittal (second row), and coronal planes (third row).

Fig. 1, 2 illustrate the qualitative comparison between the summed image generated by summing the images through frame 9–14 (15–45 min), and MVW-PC1. For qualitative comparison, the image color scale minimum and maximum levels were set to the image minimum and maximum intensity respectively. The maximum and the minimum intensity were calculated from a ROI situated on the object in the image not the background.

**Figure 2:**
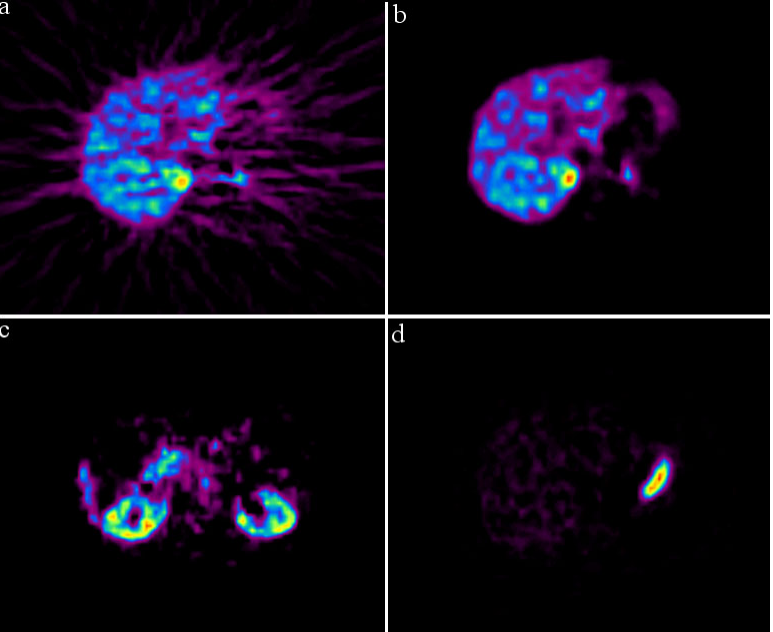
**Visual comparison between the summed image (2a), corresponding MVW-PC1 image (2b), arbitrary chosen image of MVW-PC2 (2c) and MVW-PC3 (2d)**. Notably, the MVW-PC2 and MVW-PC3 images represent tissues with two different and uncorrelated tracer pharmacokinetics compared to MVW-PC1 images. In MVW-PC2, the early pharmacokinetic events are displayed showing the parenchymal tracer accumulation in the kidneys. In MVW-PC3, the pharmacokinetic events are displayed and clearly demonstrate the [^11^C]-MTO characteristic accumulation of radioactivity in the gastric juice.

### Quantitative comparison

#### "Hot spot" method

**Figure 3:**
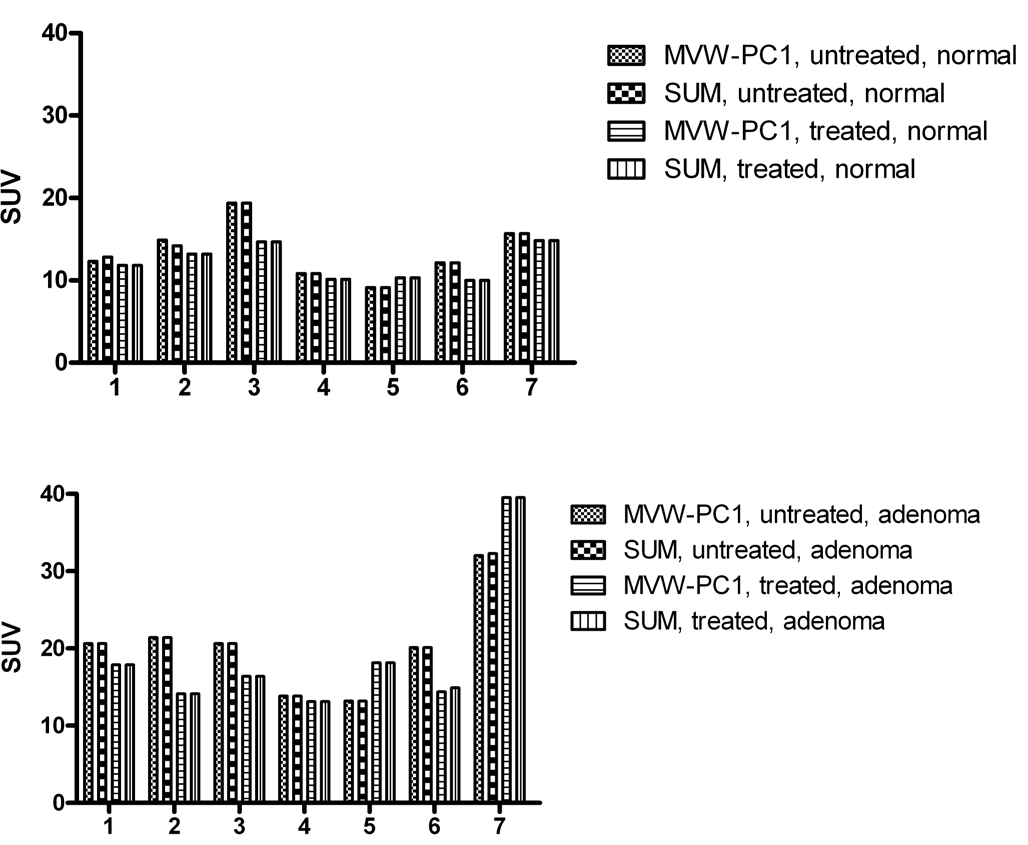
**Bar chart showing the SUV of a normal adrenal gland (upper chart) and an adrenocortical adenoma (lower chart) for each patient as obtained by using the "hot spot" method defined in both the summed and MVW-PC1 image in the MTO-PET examination in the untreated and treated state**.

**Figure 4:**
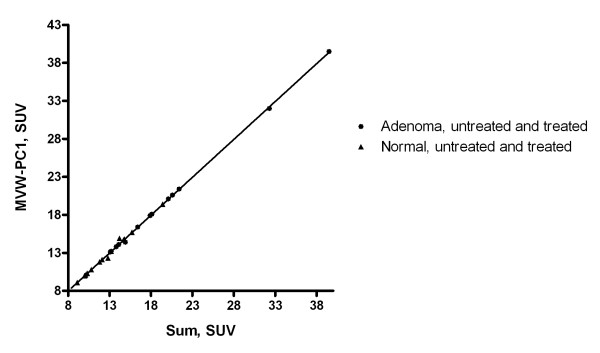
**Regression analysis of the SUVs obtained using the "hot spot" method defined in the MVW-PC1 images when plotted against the SUVs obtained using ROIs defined in the summed images shown for the adrenocortical adenomas (dots) and the normal adrenal glands (triangles)**.

When the "hot spot" method was used, the coordinates for the pixels with the highest signal in the MVW-PC1 images differed somewhat from those in the summed images (Fig. 3, Table 1). It was observed that the SUV value obtained from the adrenal adenoma from all patients was higher compared to normal adrenal when using these methods. The study on linear regression showed that the correlation between the measurements when using the two different methods, was high (Fig 4, Table 2).

**Table 1:** Results obtained from applying one-way ANOVA to the mean SUVs using the "hot spot" method, representing adrenocortical adenomas and normal adrenal glands defined in MVW-PC1 and summed images, respectively.

Comparison	Significant (P < 0.001)	Mean difference
MVW-PC1, untreat, Adenoma vs MVW-PC1, treated, adenoma	No	1.171
MVW-PC1, untreat, Adenoma vs MVW-PC1, untreated, normal	No	6.771
SUM, untreated, adenoma vs SUM, treated, adenoma	No	1.143
SUM, untreated, adenoma vs SUM, untreated, normal	No	6.843
MVW-PC1, treated, adenoma vs MVW-PC1, treated, normal	No	6.943
SUM, treated, adenoma vs SUM, treated, normal	No	7.014
MVW-PC1, untreated, normal vs MVW-PC1, treated, normal	No	1.343
SUM, untreated, normal vs SUM, treated, normal	No	1.314

**Table 2:** Parameters obtained when performing regression analysis of the SUVs obtained using the "hot spot" method defined in the MVW-PC1 images vs. the SUVs obtained using ROIs defined in the summed images.

Question	Parameters	value
Best fit value	Slope	0.9972 ± 0.001907
Best fit value	1/slope	1.003
95% Confidence Intervals	Slope	0.9931 to 1.001
Is slope significantly non-zero?	T	523
Is slope significantly non-zero?	DF	13.00
Is slope significantly non-zero?	P Value	<0.0001
Is slope significantly non-zero?	Deviation from zero?	Significant

#### Time activity curves

**Figure 5:**
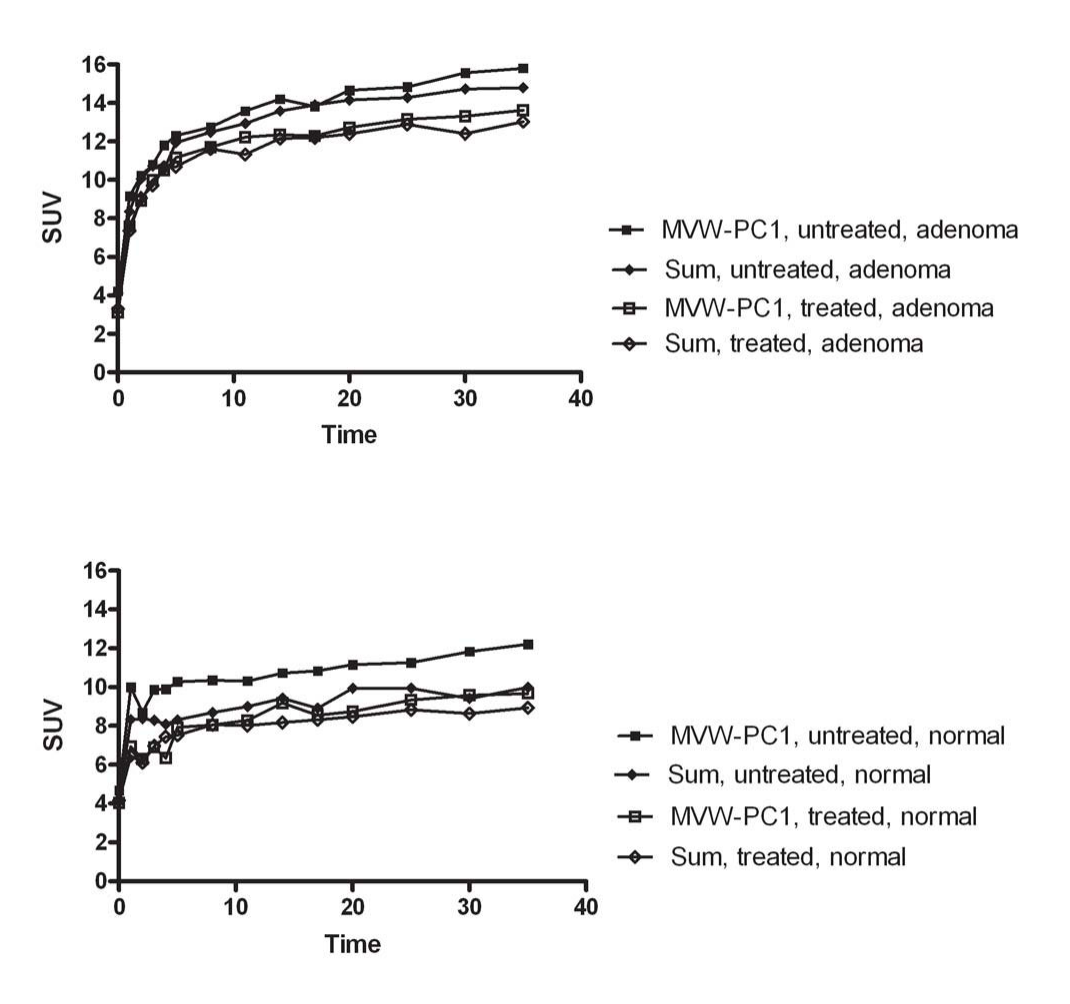
**Mean TACs of all patients (error bars have been omitted for clarity), demonstrating the pharmacokinetic behavior of [^11^C]-MTO in the adrenocortical adenomas and normal adrenal glands before and after cortisone treatment, represented by defined ROIs using the 50% cut-off method in the MVW-PCA images and summed images**.

The TACs obtained from ROIs representing adrenocortical tumours and normal adrenals demonstrated both higher mean SUVs in the untreated states than in the treated states. This was true both when MVW-PC1 was used and when the ROIs were defined in the summed images (Fig 5).

**Figure 6:**
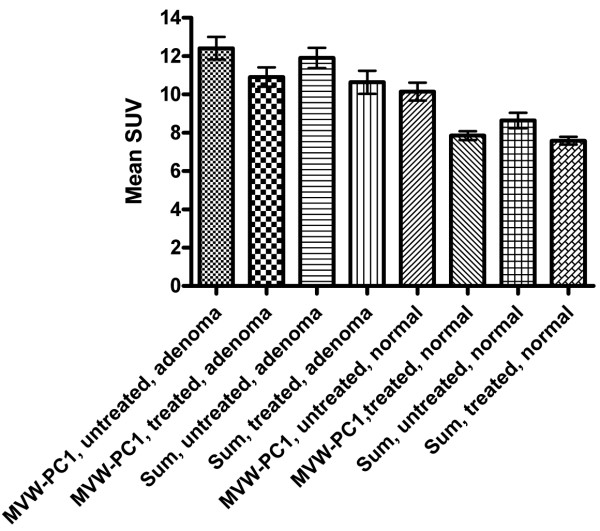
**Bar chart shows the mean SUVs of 7 patients calculated in ROIs representing the adrenocortical adenomas and normal adrenal glands**. The means are defined in both the summed and MVW-PC1 images, using the 50% cut-off method before and after cortisone treatment.

The magnitude of the mean SUV in the adrenal was higher in the untreated state than in the treated state for ROIs defined by using both the summed and the MVC-PC1 images (Fig. 6 and Table 3). Similarly, the magnitude of the mean SUV in the adrenocortical adenomas was higher in the untreated state than in the treated state for ROIs defined by using both the summed and the MVC-PC1 images (Fig. 6 and Table 4).

**Table 3:** Results obtained from applying a paired t-test on the mean SUVs calculated from ROIs (50% cut-off method) representing adrenocortical adenomas and normal adrenal glands defined in MVW-PC1 and summed images, respectively.

Comparison	Significant (P < 0.001)	Mean difference
Untreated vs. treated, MVW-PC1, normal	Yes	2.3
Untreated vs. treated, SUM, normal	No	1.054
Untreated vs. treated MVW-PCA, adenoma	Yes	1.503
Untreated vs. treated, SUM, adenoma	No	1.272

**Table 4:** Results obtained from applying a two-tailed t-test on the mean difference in percent of SUVs calculated from ROIs (50% cut-off method) representing adrenocortical adenomas and normal adrenal glands defined in MVW-PC1 and summed images, respectively.

Comparison	P value	Mean ± SEM of MVW-PC1, SUM
MVW-PC1, adenoma,% vs SUM, adenoma,%	0.2906	12.78 ± 1.258, 10.90 ± 1.213
MVW-PC1, normal,% vs SUM, normal,%	0.0002	22.59 ± 1.711, 11.91 ± 1.731

## Discussion

PCA has mostly been used for statistical analysis, data reduction and improvement of image quality of clinical PET examinations such as in the study by Pavlopoulos et al. [19]. However, these approaches are not similar to the method we used here. In our previous studies [16,17], we demonstrated that by removing the background pixels, which contained only noise, from the image field, and by performing noise pre-normalization in conjunction with volume wise application of PCA (MVW-PCA) on a preprocessed dataset, the performance of the PCA was improved. Therefore, the aim here was to apply the introduced MVW-PCA approach to MTO-PET examinations to obtain diagnostic information from the whole dataset.

Previous analyses on the metabolite spectrum in plasma have indicated rather complex conditions with a rapid metabolism of [^11^C]-MTO, leading to a dominance of metabolites in protein-free plasma, but with intact tracer bound to plasma proteins. Therefore, with a simplified approach of using a standardized summation of the image data, there is a risk that the pharmacokinetic pattern of the individual patient is ignored and potential diagnostic information may be missed.

For several PET tracers used in our department, including MTO, these time frames, 15–45 minutes, are typically summed to achieve the highest possible tumour-to-normal-tissue contrast ratio and the lowest possible image noise [8]. Compared to these summed images, MVW-PCA was not able to retrieve additional diagnostic information from the earlier and later phase of the MTO-PET examinations. By contrast, the MVW-PCA created images with improved quality and visualization of both the adrenocortical tumours and the normal adrenals (Fig. 1, 2). This was merely based on our visual comparison where the anatomical details and the image noise were assessed. The improvement in image quality is also likely to facilitate the delineation of adrenal lesions and thereby increase the precision by which ROIs are manually drawn, especially when investigating smaller tumours. Thus, MVW-PCA generates several components with various pharmacokinetic phases of the administered tracer, implying that among different adrenal tumours there may be discrepant pharmacokinetic behavior leading to easier detection and delineation in separate MVW-PCs.

In our PET examinations, MVW-PCA was applied to images reconstructed by using FBP. For decades, datasets reconstructed by FBP have formed the basis for quantitative assessment in PET research. Therefore, FBP reconstructed datasets were chosen for the initial evaluation of MVW-PCA on dynamic MTO-PET examinations, rather the Ordered Subset Expectation Maximization (OSEM) algorithm [20].

The high correlation between SUVs of the "hot-spot" ROIs defined in the summed images and those retrieved in the MVW-PC1 component indicates that the MVW-PCA technique does not introduce unexpected errors into the measurements (Fig. 4).

The results of the PET measurements by the "hot spot" ROIs (4 pixels) method were somewhat different when MVW-PCA was used compared to the summed images (Fig. 3). In several patients the coordinates of the pixels with the highest radioactivity concentration values in MVW-PC1 were shifted one pixel compared to the summed images. The number of pixels with the highest values was 5 or 6 instead of 4 due to the decreased magnitude of the noise in the MVW-PC1 images. The decrease of noise creates a lower difference of values between the neighbor pixels in MVW-PC1 images. When analyzing the summed images the pixel values are summed without correcting for the noise magnitude. However, these differences were not statistically significant.

By contrast, the TACs, i.e., 50% cut-off method (Fig. 5) of normal adrenal glands and adrenal adenomas derived from ROIs outlined in the MVW-PC1 images demonstrated a significantly higher mean SUV than those based on the summed images, being verified in both the examinations performed before and after cortisone treatment. Thus, the application of MVW-PCA leads to improved precision in defining the ROIs since pixels with the highest radioactivity concentration are included. The statistical analysis using repeated measures ANOVA (50% cut-off) showed significant differences between treated and untreated adrenocortical adenomas and normal adrenal glands when the MVW-PC1 was used whereas the summed images did not. Thus, MVW-PC1 images may provide a higher level of confidence in the statistical data in clinical trials in which PET measurements are used. Theoretically, by using MVW-PCA, either alternatively or concurrently, it may be possible to achieve the same statistical power, while reducing the number of patients in the trial. This must, however, be further evaluated.

A limitation with this study is the small number of patients in whom no dramatic impact was shown on the precision of the PET measurements by applying MVW-PCA. Moreover, in this first study applying MVW-PCA on PET of organs other than the brain, merely FBP was used. In future, work should also include automation of this method and MVW-PCA should be applied to PET examinations reconstructed by the OSEM method. Similarly, the output of the ROI analysis, including graphical illustrations, may be generated and illustrated automatically. The ongoing trend in PET tracer development to replace tracers with short half-lives, such as^11^C tracers, with longer lived tracers also includes the currently studied [^11^C]-MTO with ^18^F-labeled analogs presently being developed. These analogs will provide more time for the tracer to accumulate in adrenocortical tumours as well as be eliminated from normal tissues. However, the radiation dose to the patient will be higher than for [^11^C]-MTO, which is approximately 3 mSv.

## Conclusion

In conclusion, MVW-PCA applied on FPB reconstructed dynamic PET examinations of patients with small adrenocortcal adenomas improved the image quality by decreasing the image noise and increased the precision by which the ROIs were defined for PET measurements. The decrease in tracer accumulation induced by cortisone treatment was more evident in the TAC analysis when MVW-PCA was used as compared to when ROIs were defined in the conventionally summed images.

## Competing interests

The authors declare that they have no competing interests.

## Authors' contributions

Authors PR, JH, AM AS participated in the design, and performed the image and data analysis, and drafted the manuscript. PH and BL contributed with some of the practical approaches and the writing of the paper. All authors read and approved the final version of the manuscript.

## Pre-publication history

The pre-publication history for this paper can be accessed here:

http://www.biomedcentral.com/1471-2342/9/6/prepub

## References

[B1] Kloos R Gross MD Francis I Korobkin M Shapiro B Incidentally discovered adrenal masses Endocr Rev 1995 16 460 484 8521790 10.1210/edrv-16-4-460

[B2] Bülow B Ahrén B Adrenal incidentaloma-experience of a standardized diagnostic programme in the Swedish prospective study J Intern Med 2002 252 239 246 10.1046/j.1365-2796.2002.01028.x 12270004

[B3] NIH state-of-the-science statement on management of the clinically inapparent adrenal mass ("incidentaloma") NIH Consens State Sci Statements 2002 19 25 14768652

[B4] Grumbach M Biller B Braunstein G Campbell K Carney J Godley P Harris E Lee J Oertel Y Posner M Schlechte J Wieand H Management of the clinically inapparent adrenal mass ("incidentaloma") Ann Intern Med 2003 138 5 424 429 12614096 10.7326/0003-4819-138-5-200303040-00013

[B5] Mansmann G Lau J Balk E Rothberg M Miyachi Y Bornstein S The clinically inapparent adrenal mass: update in diagnosis and management Endocr Rev 2004 25 309 340 10.1210/er.2002-0031 15082524

[B6] Mulatero P Stowasser M Loh KC Fardella CE Gordon RD Mosso L Gomez-Sanchez CE Veglio F Young WF Jr Increased diagnosis of primary aldosteronism, including surgically correctable forms, in centers from five continents J Clin Endocrinol Metab 2004 89 1045 1050 10.1210/jc.2003-031337 15001583

[B7] Hennings J Lindhe Ö Bergström M Långström B Sundin A Hellman P [11C]-Metomidate Positron Emission Tomography of Adrenocortical Tumours in Correlation with Histopathological Findings J Clin Endocrinol Metab 2006 91 1410 1414 10.1210/jc.2005-2273 16403816

[B8] Bergström M Juhlin C Bonasera TA Sundin A Rastad J Akerstrom G Långström B PET imaging of adrenal cortical tumours with the 11β-hydroxylase tracer 11C-metomidate J Nucl Med 2000 41 275 282 10688111

[B9] Minn H Salonen A Friberg J Roivainen A Viljanen T Långsjö J Salmi J Valimäki M Nagren K Nuutila P Imaging of adrenal incidentalomas with PET using 11C-metomidate and ^18 ^F-FDG J Nucl Med 2004 45 972 979 15181132

[B10] Zettinig G Mitterhauser M Wadsak W Becherer A Pirich C Vierhapper H Niederle B Dudczak R Kletter K Positron emission tomography imaging of adrenal masses: 18F-fluorodeoxyglucose and the 11β-hydroxylase tracer 11C-metomidate Eur J Nucl Med Mol Imaging 2004 31 403 410 10.1007/s00259-004-1575-0 15197504

[B11] Hennings J Hellman P Ahlström H Sundin A Computed tomography, magnetic resonance imaging and 11C-metomidate positron emission tomography for evaluation of adrenal incidentalomas Eur J Radiol 2009 69 314 23 10.1016/j.ejrad.2007.10.024 18082990

[B12] Razifar P Lubberink M Schneider H Långström B Bengtsson E Bergström M Non-isotropic noise correlation in PET data reconstructed by FBP but not by OSEM demonstrated using autocorrelation function BMC Med Imaging 2005 5 1 15892891 10.1186/1471-2342-5-1 PMC1142517

[B13] Razifar P Sandström M Schneider H Långström B Maripuu E Bengtsson Bergström M Noise correlation in PET, CT, SPECT and PET/CT data evaluated using autocorrelation function BMC Med Imaging 2005 5 3 16122383 10.1186/1471-2342-5-3 PMC1208889

[B14] Zaidi H Montandon M-L Meikle SR Strategies for attenuation compensation in neurological PET studies Neuroimage 2007 34 518 541 10.1016/j.neuroimage.2006.10.002 17113312

[B15] Boellaard R Krak NC Hoekstra OS Lammertsma AA Effects of noise, image resolution, and ROI definition on the accuracy of standard uptake values: a simulation study J Nucl Med 2004 45 1519 1527 15347719

[B16] Razifar P Axelsson J Schneider H Långström B Bengtsson E Bergström M A new Application of Pre-normalized Principal Component Analysis for Improvement of Image Quality and Clinical Diagnosis in Human Brain PET Studies – Clinical brain studies using [11C]-GR20 [11C]-L-deuterium-deprenyl, [11C]-5-Hydroxy-L-Tryptophan, [11C]-L-DOPA and Pittsburgh Compound-B Neuroimage 5171 33 588 598 10.1016/j.neuroimage.2006.05.060 16934493

[B17] Razifar P Axelsson J Schneider H Långström B Bengtsson E Bergström M Volume-Wise Application of Principal Component Analysis on Masked Dynamic PET Data in Sinogram Domain IEEE Tans Nucl Sci 2006 53 2759 2768 10.1109/TNS.2006.878008

[B18] Sundin A Johansson C Hellman P Bergström M Ahlström H Jacobson GB Långström B Rastad J PET and parathyroid L-[carbon-11]methionine accumulation in hyperparathyroidism J Nucl Med 1996 37 1766 70 8917171

[B19] Pavlopoulos S Thireou T Kontaxakis G Santos A Analysis and interpretation of dynamic FDG PET oncological studies using data reduction techniques Biomed Eng Online 2007 6 36 17915012 10.1186/1475-925X-6-36 PMC2228305

[B20] Hudson HM Larkin RS Accelerated image reconstruction using Ordered Subsets of Projection data IEEE Trans Med Imaging 1994 13 601 609 10.1109/42.363108 18218538

